# Myeloid PTEN loss affects the therapeutic response by promoting stress granule assembly and impairing phagocytosis by macrophages in breast cancer

**DOI:** 10.1038/s41420-024-02094-0

**Published:** 2024-07-30

**Authors:** Yan Li, Chao Xu, Xiaojun Qian, Gang Wang, Chaoqiang Han, Hui Hua, Menghao Dong, Jian Chen, Haiyang Yu, Rutong Zhang, Xiaoxi Feng, Zhenye Yang, Yueyin Pan

**Affiliations:** 1 Department of Clinical Oncology, The First Affiliated Hospital of USTC, Division of Life Sciences and Medicine, University of Science and Technology of China, Hefei, Anhui 230001 China; 2 Division of Life Sciences and Medicine, University of Science and Technology of China, Hefei, Anhui 230001 China

**Keywords:** Cancer microenvironment, Mechanisms of disease

## Abstract

Breast cancer (BRCA) has become the most common type of cancer in women. Improving the therapeutic response remains a challenge. Phosphatase and tensin homologue deleted on chromosome 10 (PTEN) is a classic tumour suppressor with emerging new functions discovered in recent years, and myeloid PTEN loss has been reported to impair antitumour immunity. In this study, we revealed a novel mechanism by which myeloid PTEN potentially affects antitumour immunity in BRCA. We detected accelerated stress granule (SG) assembly under oxidative stress in PTEN-deficient bone marrow-derived macrophages (BMDMs) through the EGR1-promoted upregulation of TIAL1 transcription. PI3K/AKT/mTOR (PAM) pathway activation also promoted SG formation. ATP consumption during SG assembly in BMDMs impaired the phagocytic ability of 4T1 cells, potentially contributing to the disruption of antitumour immunity. In a BRCA neoadjuvant cohort, we observed a poorer response in myeloid PTEN^low^ patients with G3BP1 aggregating as SGs in CD68+ cells, a finding that was consistent with the observation in our study that PTEN-deficient macrophages tended to more readily assemble SGs with impaired phagocytosis. Our results revealed the unconventional impact of SGs on BMDMs and might provide new perspectives on drug resistance and therapeutic strategies for the treatment of BRCA patients.

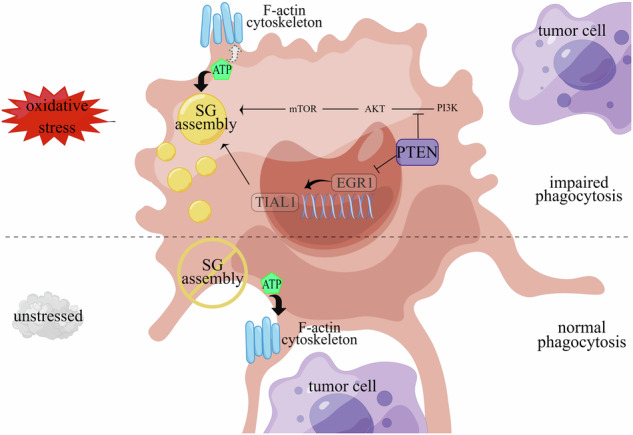

## Background

As the most prevalent type of tumour worldwide [1], breast cancer (BRCA) is known for its heterogeneity, especially its tumour microenvironment (TME) complexity [2]. Even with recent advances in oncology, many patients still suffer from poor responses and resistance to antitumour treatments. The integrated stress response (ISR) [3, 4] is an evolutionarily conserved mechanism through which cells adapt to the TME under comprehensive stress conditions, including hypoxia, ROS, DNA damage and inflammation, among which oxidative stress is considered the leading stress generated by tumour cells, immune cells and even exogenous therapeutic drugs [5–8]. As a crucial member of the TME, macrophages, especially when they are dysfunctional, play a vital role in the establishment of an environment conducive for the occurrence and metastasis of BRCA, and such environments are closely related to the tumour response to nutrient/oxygen deprivation or therapeutic drugs [8, 9]. PTEN is a canonical gene with important regulatory functions both in tumours and immune cells. Studies have shown that myeloid-related PTEN loss inhibits antitumour immunity in breast cancer patients, revealing new functions of PTEN in macrophages and the TME [10].

Like in tumour cells, macrophages assemble stress granules (SGs) as protection when facing stress [11–15]. As a kind of nonmembrane cell organelle and product of liquid–liquid phase separation (LLPS), SGs protect tumour cells from death, contributing to tumour resistance [16–18]; however, the role and regulatory mechanism of SGs in immune cells remain unclear and require additional research.

In the tissue sections from a cohort of patients with BRCA who received neoadjuvant treatment, we identified SGs in macrophages from patients with low myeloid PTEN expression who were also relatively insensitive to treatment. By inducing SGs in BMDMs, we observed impaired phagocytosis of 4T1 cells accompanied by marked ATP consumption in macrophages. Moreover, PTEN-deficient macrophages were more likely to form SGs due to the upregulation of the expression of the nucleating protein TIAL1 and the activation of the PAM pathway, and supplementation with ATP or PAM inhibition could reverse the damage caused by BMDM phagocytosis after SG assembly. Our study explored the impact of SGs on the antitumour immunity of BMDMs and revealed an unexpected role of PTEN in the TME, thus providing a new outlook for BRCA treatment, especially for patients with myeloid PTEN dysfunction.

## Results

### Myeloid PTEN is related to SGs in macrophages and the antitumour immune response in BRCA patients

PTEN inactivation is the most common type of tumour suppressor dysfunction in human cancers, occurring in approximately 40% of BRCA patients, especially in patients with triple-negative breast cancer (TNBC) [19]. PTEN status not only affects the behaviour of cancer cells but also influences the microenvironment directly or indirectly by regulating immune responses [20, 21]. PTEN dysfunction of in different immune cells shifts the global balance of the TME towards immunosuppression [22, 23].

**Fig. 1:**
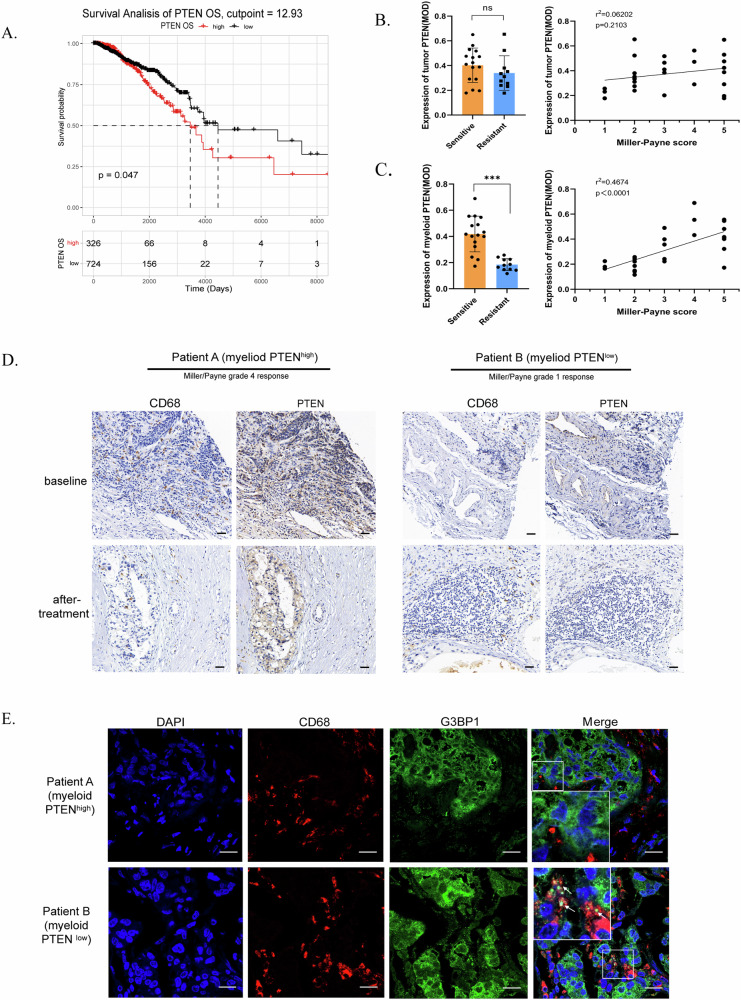
Myeloid PTEN is related to SGs in macrophages and the antitumour immune response in BRCA patients. **A** Survival curve for patients grouped according to the optimal cut-off for PTEN expression. The cut-off points used are displayed in Supplementary Fig. 1A and 1B. **B** Analyses of the correlation between therapeutic sensitivity and tumour PTEN expression. *n* = 27 patients (16 patients in the sensitive group and 11 in the resistant group based on the Miller–Payne grading system). PTEN and G3BP1 expression in CD68-positive macrophages was assessed by the MOD (immunohistochemical modification) value (Supplementary Table 2). For the histograms, the data are presented as means ± SDs; p values were determined by t tests; asterisks indicate significant differences from the control; * *p* < 0.05, ** *p* < 0.01, *** *p* < 0.001. For the correlation curves, the R^2^ and *p* value were determined by linear regression analysis. **C** Analyses of the correlation between therapeutic sensitivity and myeloid PTEN expression. **D** IHC staining of CD68 and PTEN in tissue sections from BRCA patients in the neoadjuvant treatment cohort. Macrophages were identified as CD68-positive cells. The scale bar is 25 μm. **E** Immunofluorescence staining of G3BP1 and CD68 in sections from the BRCA cohort. The macrophages were labelled with CD68 (red); arrowheads indicating G3BP1 (green) aggregation indicate the formation of SGs. The scale bar is 10 μm.

Although PTEN is regarded as a tumour suppressor, high PTEN expression in general tissues was not indicative of improved survival among BRCA patients (Fig. 1A, Supplementary Fig. 1A & 1B), as determined by our analysis of patient data from the TCGA-BRCA database. We subsequently measured PTEN expression in tumour cells and stromal cells separately and explored whether there were distinct links between PTEN levels and therapeutic outcomes. We collected clinical data and pathological sections from patients with BRCA who received neoadjuvant chemotherapy (anthracycline- and taxane-based regimens) at our hospital and evaluated treatment efficacy via the Miller–Payne (MP) scoring system [24] (Supplementary Table 1). Patients with 1 ~ 2 MP points were defined as having therapeutic resistance, whereas those with 3 ~ 5 MP points were defined as having therapeutic sensitivity. By immunohistochemical (IHC) staining and MOD measurements, we did not observe an explicit correlation between PTEN expression in tumour cells and the MP score (Fig. 1B). However, we found that PTEN expression in CD68-positive cells was lower in patients with drug resistance and that patients who had higher myeloid PTEN expression tended to be more sensitive to treatment, revealing a significant correlation between myeloid PTEN expression and MP scores (Fig. 1C & D). Our previous work revealed that myeloid PTEN promoted antitumour immunity by accelerating NLRP3 inflammasome activation [10] and that inflammasomes and SGs were also connected as diverging pathways of cell fate in stress conditions [15]. Therefore, we asked whether SGs are also involved in the regulation of the TME by myeloid PTEN status.

Cells assemble SGs for self-protection under stress; in the TME, oxidative stress is the primary stress stimulus. Therefore, we first evaluated whether the response to oxidative stress affects clinical outcomes. Using patient data from the TCGA-BRCA database, we obtained gene sets related to positive or negative regulation of the response to oxidative stress (GO:1902884 and GO:1902883) from the GO knowledgebase for enrichment analysis and then classified the patients into high-ES and low-ES groups according to the ES cut-off value. The results indicated that samples in the positive_regulation_high subgroup had a worse prognosis (Supplementary Fig. 1C), as indicated by the negative correlation between genes that are positively related to the response to oxidative stress and the prognosis of BRCA patients. The negative_regulation_of_response_to_oxidative_stress gene set showed no significant difference in OS (Supplementary Fig. 1,D). To explore our hypothesis, we attempted to identify clinical evidence of SGs in macrophages from BRCA patients and explored the relevance of SGs to the clinical outcomes of patients with BRCA. By immunofluorescence staining of G3BP1 in pathological sections from the BRCA cohort, we found that G3BP1 was distributed as rough specks and even as droplet aggregates in CD68-positive cells in several myeloid PTEN^low^ samples, indicating the formation of SGs. In myeloid PTEN^high^ samples, G3BP1 staining revealed a fine sand-like distribution in the cytoplasm with no droplet aggregates (Fig. 1E). These findings indicated that SG formation might occur more readily in PTEN-deficient macrophages and might participate in the drug resistance mechanism in BRCA patients with corresponding myeloid PTEN statuses.

### SG assembly was accelerated in PTEN-deficient BMDMs

**Fig. 2:**
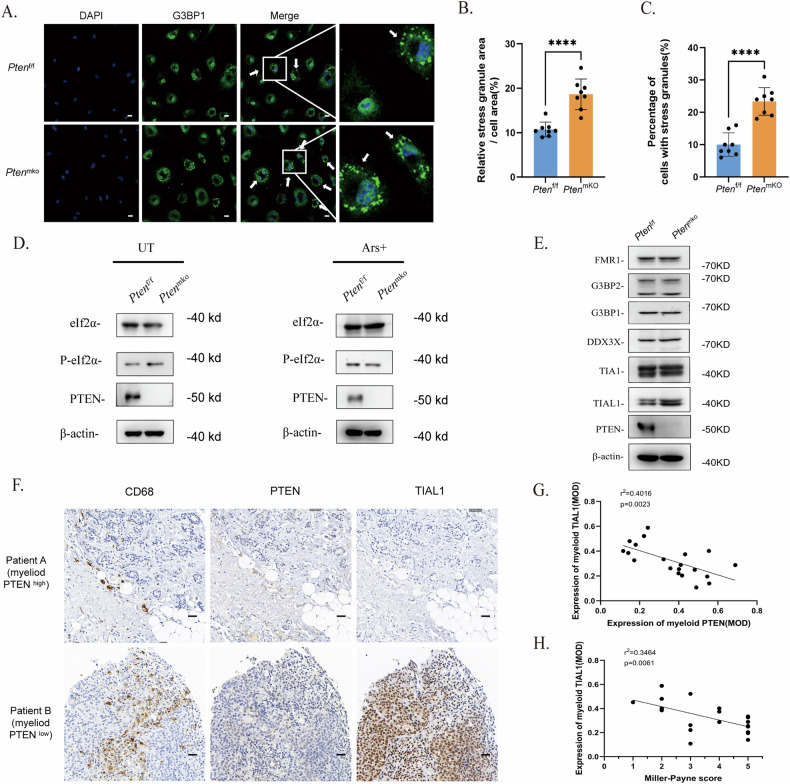
SG assembly is accelerated in PTEN-deficient BMDMs. **A** Immunofluorescence staining of SGs (indicated by the aggregation of G3BP1) in BMDMs from *Pten*^f/f^ or *Pten*^mKO^ mice. BMDMs were stimulated with LPS (100 ng/mL) for 4 h and induced with arsenite (90 μM) for 1 h. G3BP1 was labelled with Alexa 488. The scale bar is 10 μm. **B** and **C** SG counts in BMDMs. The data are presented as means ± SDs; *n* = 3 independent experiments, and p values were determined by t tests. **D** Protein levels of eIF2α and P-eIF2α in BMDMs. **E** The protein levels of several conventional nucleating proteins in BMDMs without pretreatment. **F** Immunohistochemical staining for CD68, PTEN and TIAL1 in patients with different myeloid PTEN statuses. The scale bar represents 25 μm. **G** Correlation analysis between the MOD of myeloid PTEN and myeloid TIAL1 in the BRCA cohort. **H** Correlation analysis between the MOD of myeloid TIAL1 and the Miller–Payne score in the BRCA cohort.

According to the evidence of SGs in macrophages in myeloid PTEN^low^ BRCA samples, we presumed that PTEN-deficient macrophages might tend to form SGs more easily. For confirmation, we induced SGs via arsenite (Ars) in BMDMs from *Lys2*-Cre-*Pten*^f/f^ (*Pten*^mKO^) and *Pten*^f/f^ mice and assessed the accumulation of SGs in *Pten*^mKO^ BMDMs (Fig. 2A & B). Considering that conventional SG assembly is triggered by the phosphorylation of eukaryotic translation initiation factor 2 subunit alpha (EIF2S1, eIF2α), we assessed the phospholipid esterase activity of PTEN in the two groups of macrophages. However, no significant difference in the protein level of eIF2α or P-eIF2α was found between *Pten*^f/f^ and *Pten*^mKO^ BMDMs, indicating that eIF2α phosphorylation was not associated with PTEN status in BMDMs (Fig. 2C).

As SGs are a product of LLPS in the cytoplasm, their formation is also driven by elevated concentrations of nucleating component proteins [25, 26]. Next, we measured the protein levels of several key RNA-binding proteins, such as G3BP, TIA1/TIAL1 and DDX3X, in SGs and found that TIA1/TIAL1, especially TIAL1, was highly expressed (Fig. 2E). Consistently, in the cohort of BRCA patients who received neoadjuvant chemotherapy, IHC staining of BRCA tissues also revealed higher TIAL1 expression in myeloid PTEN^low^ samples than in myeloid PTEN^normal^ or myeloid PTEN^high^ samples (Fig. 2F & G). Like that of myeloid PTEN, the expression of myeloid TIAL1 was correlated with therapeutic efficacy in BRCA patients.

### PTEN deficiency is accompanied by upregulated EGR1 expression in BMDMs

**Fig. 3:**
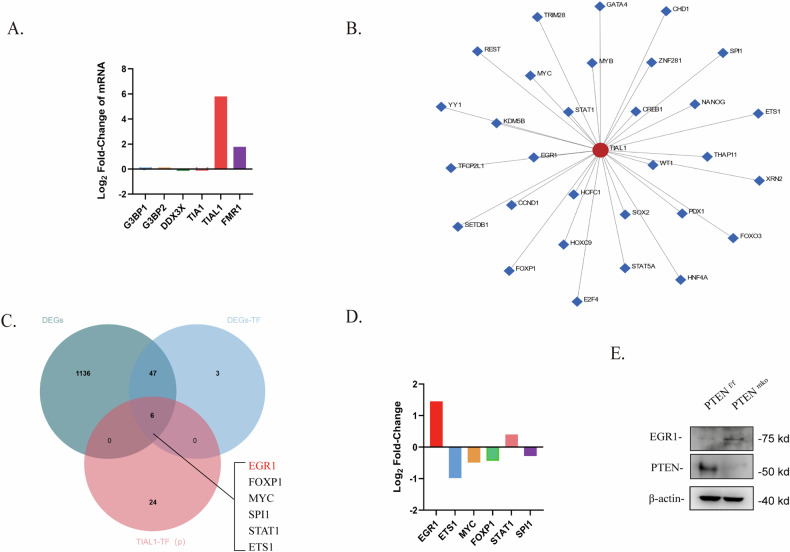
PTEN deficiency is accompanied by upregulated EGR1 expression in BMDMs. **A** Rank of the log2-fold change in the expression of several conventional SG proteins in BMDMs from *Pten*^f/f^ or *Pten*^mko^ mice based on the RNA-seq results. **B** Transcription factor prediction for the potential regulation of TIAL1. **C** Venn diagram of the predicted TFs of TIAL1 in **B** and the total TFs among the DEGs in BMDMs from *Pten*^f/f^ or *Pten*^mko^ mice. **D** Ranking of log2-fold changes among the 6 potential TFs obtained in **D** based on the RNA-seq results. **E** Protein level of EGR1 in BMDMs from *Pten*^f/f^ and *Pten*^mko^ mice without pretreatment.

To investigate the mechanism underlying the upregulation of TIAL1 expression by PTEN loss in BMDMs, we performed RNA-seq with BMDMs from *Pten*^f/f^ or *Pten*^mko^ mice and identified the differentially expressed genes (DEGs). The mRNA levels of the aforementioned SG component proteins were evaluated, and the mRNA expression of TIAL1 was the most significantly upregulated (Fig. 3A), suggesting that the transcription of TIAL1 is altered in Pten-deficient BMDMs. We predicted the transcription factors (TFs) of TIAL1 using ChEA software and the ChIP-X database [27] (Fig. 3B). Moreover, we screened out all the TFs among the DEGs between *Pten*^f/f^ and *Pten*^mko^ BMDMs (Supplementary Table 3) and obtained genes that intersected with the predicted TFs. Finally, 6 potential TFs of TIAL1 were identified in BMDMs (Fig. 3C & Supplementary Table 3). We evaluated the differences in the expression of the 6 genes in the RNA sequencing data and found that EGR1 was the only gene with a |Log2FC| greater than 1 (Fig. 3D), which suggested that it was the most likely gene that might upregulate TIAL1 transcription in BMDMs. For verification, we measured the protein level of EGR1 in BMDMs from *Pten*^f/f^ and *Pten*^mko^ mice and found that EGR1 expression significantly increased after PTEN deletion (Fig. 3E).

### Upregulated EGR1 promoted the transcription of TIAL1 in PTEN-deficient BMDMs

**Fig. 4:**
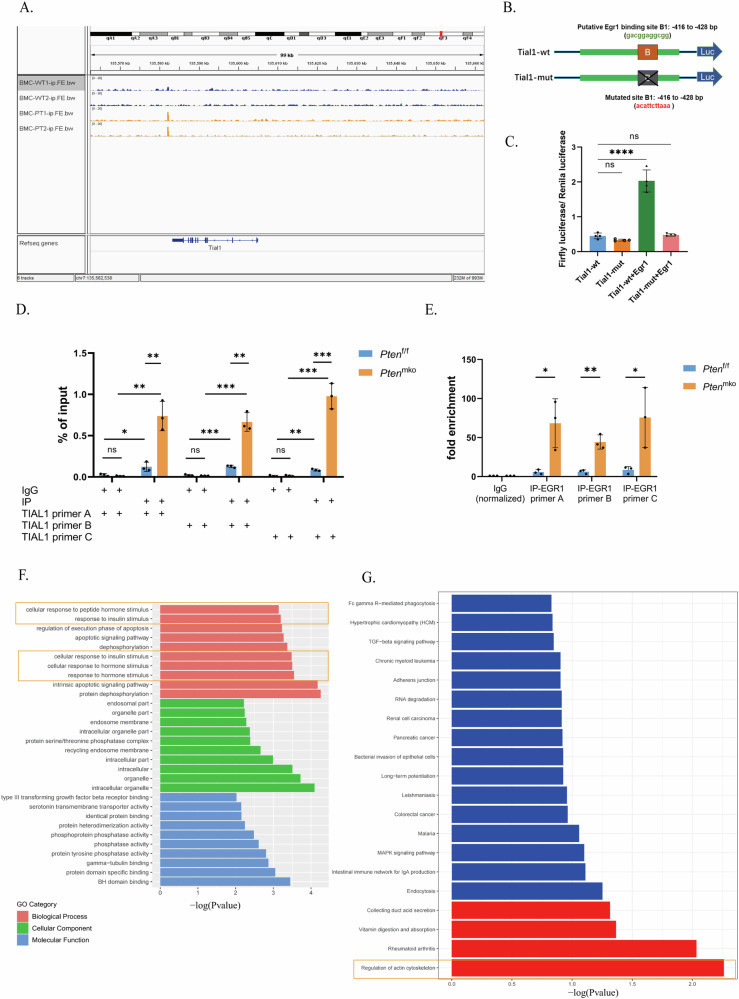
Upregulated EGR1 promotes the transcription of TIAL1 in PTEN-deficient BMDMs. **A** ChIP-seq of EGR1 in BMDMs from *Pten*^f/f^ and *Pten*^mko^ mice. **B** Diagram of plasmid construction. Wild-type (TIAL1-wt) and Egr1 binding region-mutated (TIAL1-mut) plasmids harbouring the TIAL1 promoter reporter gene were constructed using the PGL3.0 vector. **C** Dual-luciferase reporter gene assay of TIAL1 and EGR1. **D** and **E** ChIP-qpcr analyses, including % of input (**D**) and fold enrichment (**F**) using the EGR1 antibody and 3 different primers of TIAL1. **F, G** KEGG and GO enrichment analyses of peak-related genes identified via ChIP-seq analysis of EGR1 in BMDMs from *Pten*^f/f^ and *Pten*^mko^ mice.

To investigate the transcriptional relationship between EGR1 and TIAL1, EGR1 ChIP-seq was performed with BMDMs from *Pten*^f/f^ and *Pten*^mko^ mice, and the TIAL1 peak was mapped to unique reads of the *Pten*^mko^ group (Fig. 4A), indicating that TIAL1 might be the target gene of EGR1. This finding suggested that after PTEN loss, EGR1 might bind to the TIAL1 gene and regulate its transcription in BMDMs. We further clarified the regulatory effect of EGR1 on TIAL1 via a dual-luciferase reporter gene assay. Using JASPAR software, we found potential binding sites (5’- gacgggcgg-3’) in TIAL1 located at TSSs -416 to -428 bp (Supplementary Table 5); then, using the PGL3.0 vector, we constructed wild-type (TIAL1-wt) and EGR1 binding region mutated (TIAL1-mut) plasmids harbouring the TIAL1 promoter-reporter gene (Fig. 4B). After the cotransfection with an EGR1 overexpression plasmid, the fluorescence intensity was measured. It was showed that the fluorescence was significantly enhanced in the TIAL1-mut group (Fig. 4C). For further verification, ChIP-qpcr analyses was conducted in BMDMs from *Pten*^f/f^ and *Pten*^mko^ mice using EGR1 antibody, showing amplified enrichment of TIAL1 in the *Pten*^mko^ groups (Figs. 4D and E). These results indicated that EGR1 is a transcription factor of TIAL1, and the transcription of TIAL1 was promoted by upregulated EGR1 in PTEN-deficient BMDMs. In addition, we noticed that the GO and KEGG enrichment analyses based on the ChIP-seq results indicated enriched pathways of “response to stimuli” (Fig. 3F) and “actin cell skeleton” (Fig. 3G), suggesting potential influences on cell response and function of the two groups of BMDMs .

### SG formation impaired the phagocytosis of BMDMs through marked ATP consumption

**Fig. 5:**
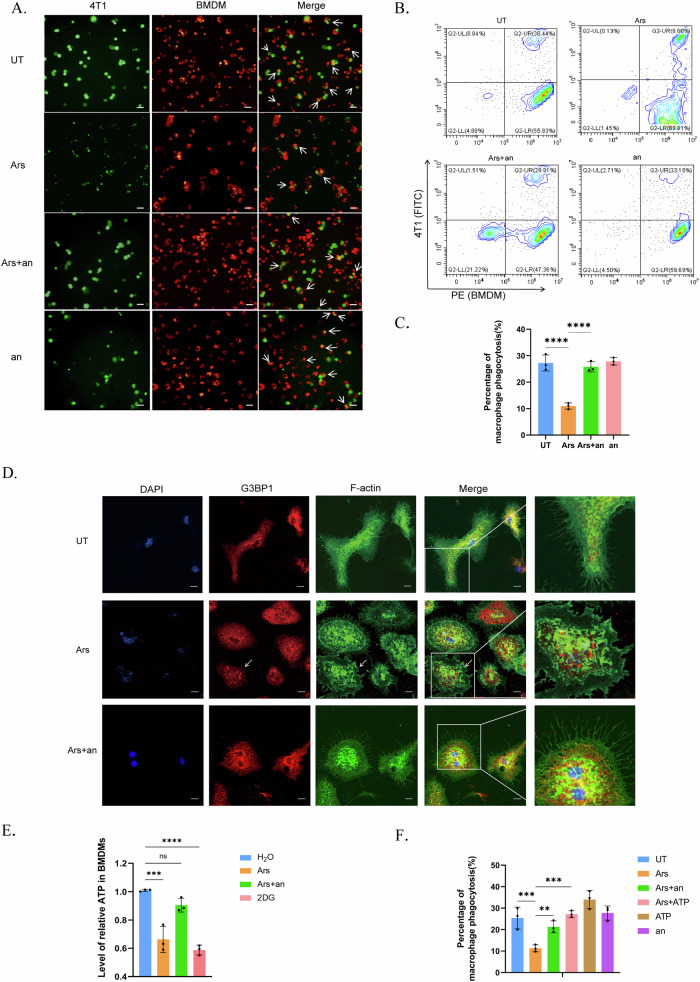
ATP consumption by SG assembly impairs phagocytosis by macrophages. BMDMs were stimulated with LPS (100 ng/mL) for 4 h. SGs were induced by arsenite (90 μM) for 1 h and inhibited by anisomycin (25 µg/ml) for 20 min before arsenite treatment. **A**~**C** Coculture of BMDMs and 4T1 cells to assess phagocytosis. BMDMs were labelled with Cell Tracking Dye Kit-Red-Cytopainter, and 4T1 cells were labelled with CFSE. BMDMs and 4T1 cells were cultured at a ratio of 5:1 for 4 ~ 6 h. Cells in **A** (indicated by white arrows) or in **B** were defined as BMDMs that underwent phagocytosis. The scale bar is 50 μm. *n* = 3 independent experiments. The data are presented as means ± SDs; p values were determined by single factor analysis of variance. **D** Fluorescence of the cytoskeleton and stress granules. The Actin-Tracker Green probe was used to label myosin filaments (F-actin), and Alexa Fluor 594 was used to label G3BP1. The aggregation of G3BP1 indicates the formation of SGs. The arrow indicates cells with SGs. The scale bar represents 10 μm. **E** Intracellular ATP levels in BMDMs. Cells were treated with 5 mM 2DG for 1 h. The data are presented as means ± SDs; *n* = 10 wells and 3 independent experiments; the *p* value was determined by two-tailed unpaired Student’s t test and a multiple comparisons test. **F** Evaluation of the phagocytosis efficiency of BMDMs towards 4T1 cells. ATP was added at 4 M for 90 min. BMDMs were labelled with Cell Tracking Dye Kit-Red-Cytopainter, and 4T1 cells were labelled with CFSE. The cells were cocultured for 5 h. The data are presented as means ± SDs; *n* = 3 independent experiments; the p value was determined by a two-tailed unpaired Student’s t test and a multiple comparisons test.

Since we discovered evidence of SGs in CD68+ cells from myeloid PTEN^low^ BRCA patients who were more treatment resistant but not from myeloid PTEN^high^ patients, we presumed that despite providing protection for survival, SGs might be detrimental to the antitumour function of BMDMs. First, we assessed several conventional inflammatory cytokines (TNF-α, IL-6 and MCP-1) in the cell culture media of BMDMs and did not observe significant differences after SG induction (Supplementary Fig. 2A), a finding that is consistent with previous results [15]. Moreover, regarding antigen presentation, the expression of MHC-II molecules on BMDMs did not significantly change (Supplementary Fig. 2C & D). However, after coculture with 4T1 cells, we found an apparent reduction in phagocytic efficiency after inducing SGs in BMDMs, an effect that could be rescued by anisomycin, a compound that has been proven to inhibit SG assembly [15] (Fig. 5A~C).

Phagocytosis is regulated by multiple processes, including receptor recognition on the membrane surface, and remodelling of cytoskeletons composed of myosin filaments, and is dependent on ATP throughout the whole process [28–31]. The regulatory receptors include “eat me” and “don’t eat me” receptors, which promote or inhibit, respectively, phagocytosis by macrophages [32]. However, we did not observe a significant difference in the mRNA levels of major regulatory receptors after SG induction (Supplementary Fig. 2,E). Biophysically, phagocytosis also requires cytoskeletal remodelling or the formation of plasma membrane protrusions (such as filopodia or pleats), which are generated by the polymerization of F-actin and are used for targeted detection. The inhibition of cytoskeleton remodelling or F-actin blockade hinders the formation of filopodia and impedes phagocytosis in BMDMs [33]. Therefore, we labelled F-actin to visualize filopodia and found that the filopodia in untreated BMDMs were densely elongated and sharp; in contrast, the cell membranes of arsenite-treated BMDMs were more flat with shortened and blunted filopodia, especially in cells with SGs (Fig. 5D). The filopodia of cells pretreated with anisomycin were similar to those of the control group. This suggested that SG formation in macrophages might hinder cytoskeletal remodelling and obstruct the extension of filopodia, which results in impaired phagocytosis.

Next, we sought to identify the cause of cytoskeletal remodelling failure. Since myosin polymerization and myosin motor proteins are indispensable for phagocytosis [31] and since some myosin subunits have been observed inside RNA granules [34], it was possible that some myosin proteins were dysfunctional because they were within SGs. Although structure prediction revealed IDRs in some myosin isoforms (Supplementary Fig. 2F), immunofluorescence staining with a pan-myosin antibody did not reveal colocalization with G3BP1 (Supplementary Fig. 2,H). Nearly all stages of cytoskeleton remodelling require ATP for energy, and macrophage phagocytosis can be inhibited by ATP deprivation [33]; furthermore, SG assembly and disassembly are both ATP-dependent processes [35]. Therefore, we measured the intracellular ATP concentration in BMDMs. ATP levels were significantly lower in BMDMs after the induction of SG assembly than in untreated cells, and the inhibition of SG assembly reversed ATP consumption (Fig. 5E). Interestingly, in contrast to what has been observed in tumours, SG induction did not reduce ATP levels in 4T1 cells (Supplementary Fig. 2G), a finding that is similar to the results of some published studies [36]. We hypothesized that when facing stress, massive amounts of ATP supply SG assembly to ensure survival, while insufficient ATP is left for cytoskeleton remodelling, target capture and phagocytosis, which are nonessential vital activities for survival. For confirmation, we replenished ATP in BMDMs after SG induction and observed rescued phagocytosis in 4T1 cells, a finding that is similar to what was observed in the SG inhibition group (Fig. 5F). These results indicated that ATP consumption by SG assembly impaired phagocytosis by macrophages.

### ATP supplementation or PAM pathway inhibition alleviated impaired phagocytosis by PTEN-deficient BMDMs

**Fig. 6:**
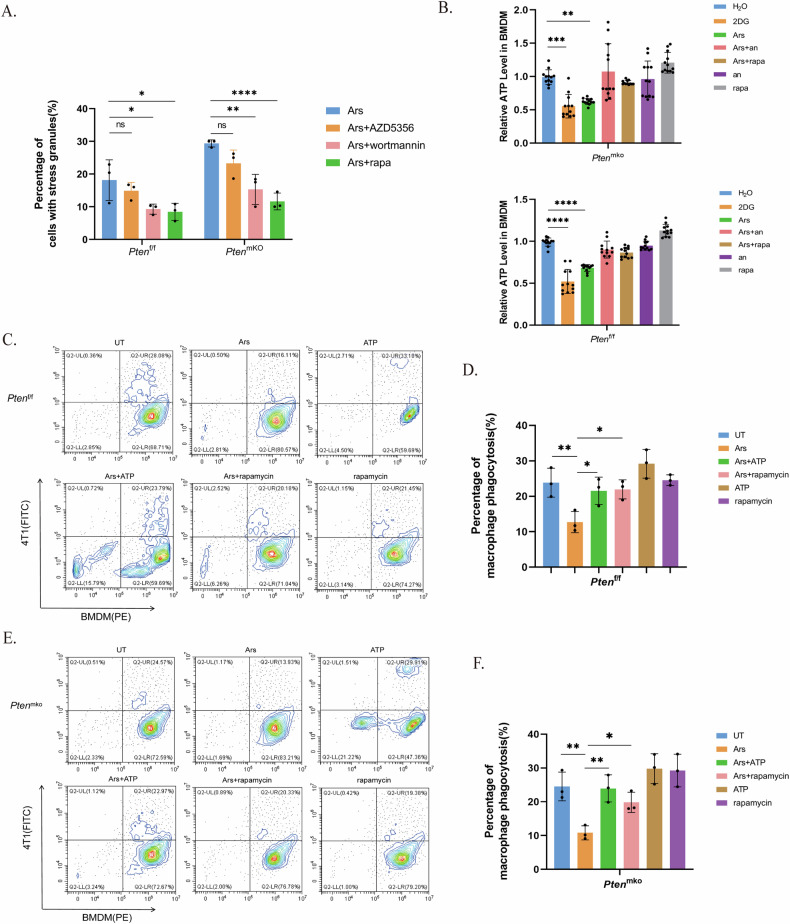
Phagocytosis impairment by SGs in PTEN-deficient BMDMs is alleviated by ATP supplementation or PAM pathway inhibition. BMDMs were stimulated with LPS (100 ng/mL) for 4 hours. SGs were induced with arsenite (90 μM) for 1 h; the cells were treated with wortmannin (100 nM), rapamycin (100 nM) or AZD5356 (3 μM) 2 h before SG induction. **A** Quantification of SGs in BMDMs treated with a PAM pathway inhibitor. The p value was determined by one-way ANOVA; The data are presented as means ± SDs; n = 3 independent experiments. **B** Intracellular ATP levels in BMDMs from *Pten*^f/f^ or *Pten*^mko^ mice. The cells were treated with 2DG (5 mM) for 1 h. ATP (4 M) was added for 90 min. The p value was determined by one-way ANOVA; the data are presented as means ± SDs; *n* = 12 wells and 4 independent experiments in each group. **C** and **D** Phagocytosis efficiency of BMDMs in *Pten*^f/f^ mice. **E** and **F** Phagocytosis efficiency of BMDMs in *Pten*^mKO^ mice.

As a vital tumour suppressor, PTEN participates in various signalling pathways that regulate cell growth and proliferation, the most notable of which is the PI3K-AKT-mTOR (PAM) pathway [37]. Studies have shown that in tumour cells, PI3K and mTOR are involved in the regulation of SG formation [38, 39]. Notably, the relationship between mTOR-related pathways and SGs varies among different cell types or under different stress conditions, and the specific impact of the mTOR pathway on SG assembly remains controversial [40]. To determine the role of the PAM pathway in SG formation in BMDMs, we treated cells with a PI3K inhibitor, an mTOR inhibitor, or a pan-AKT inhibitor before SG induction. All three inhibitors, especially the PI3K inhibitor and mTOR inhibitor, inhibited SG formation to a certain extent, which significantly decreased the difference between cells with different PTEN statuses (Fig. 6A). PAM activation by PTEN deficiency also contributed to promoting SG assembly. To further confirm the relationships among the PAM pathway, SGs and phagocytosis, we cocultured BMDMs and 4T1 cells and assessed the intracellular ATP concentration and phagocyte efficiency (Fig. 6B). The results showed increased ATP consumption and further weakening of the phagocytic function of BMDMs with PTEN deficiency, effects that could be rescued by ATP replenishment or pretreatment with rapamycin (Fig. 6C–F).

## Discussion

The TME has always been a topic of interest in research on the diagnosis and treatment of BRCA. A high level of immune infiltration has been confirmed to be associated with an increased immune response and improved survival in some subtypes of BRCA, but results pertaining to the pCR rate after neoadjuvant chemotherapy have been inconsistent [41], indicating that further exploration is needed. As a major cell population in the TME, macrophages play a vital role in innate immunity against tumours. SG formation is a conservative self-protective mechanism for eukaryotic cells in mammals that promotes cell survival in adverse environments and is thus closely related to therapeutic resistance in cancer cells [14, 17, 18, 42–44]. SGs generated by immune cells reportedly assist survival [15], but the impact of SGs on immune functions has not been fully elucidated. Our study revealed that, in macrophages, this survival strategy might sacrifice the antitumour capacity of these cells, which contributes to drug resistance in BRCA patients. Our research might provide new perspectives on the mechanisms of macrophage dysfunction, especially in patients with PTEN deficiency.

SGs are dynamic, reversible cellular organelles formed by concentrated stress proteins and nucleic acids in eukaryotic cells [13, 45, 46]. Both the assembly and disassembly of SGs are ATP-dependent processes [35, 47], and ATP-dependent proteins/nucleic acids are present in yeast and mammalian SGs; these proteins include protein partners (such as the Hsp70/Hsp40 and CCT complex) and various RNA and DNA unwindases [35]. This might explain the noticeable ATP consumption by SGs in macrophages and the subsequent obstruction of cellular skeleton remodelling in our study, effects that could be rescued by ATP replenishment. Interestingly, we observed the opposite trend, i.e., ATP levels did not change in 4T1 cells after arsenite treatment and even increased compared to those in untreated cells, a finding that is consistent with published results of study on cancer cells [36]. We presumed that this difference may be associated with the high metabolic character of tumour cells, which enable more efficient ATP production, even in the case of stress and SG assembly; moreover, the potential alternative pathways retained for ATP generation also maintain malignant biological features in immortal cells. This difference might explain the difference in the impact of SG assembly on the intracellular ATP concentration between tumour cells and BMDMs, which deserves further exploration in future studies.

Classic SG formation is initiated by the activation of four kinase families of stress sensors in response to adverse environmental stimuli: HRI (heme-regulated initiation factor 2α kinase), PKR (interferon-inducible double-stranded RNA-dependent protein kinase), PERK (protein kinase R-like ER kinase), and GCN2 (general control nonderepressible 2) [48–52]. The subsequent phosphorylation of eIF2α causes protein translation arrest, and the aggregation of biomolecules (such as mRNPs) eventually leads to SG assembly [11, 53]. Therefore, multiple regulatory factors could affect SG formation, such as the phosphorylation of eIF2α, interactions between biomolecules, and the plasma concentration of nucleating proteins with RNA-specific binding domains. In our study, PTEN loss did not accelerate SG formation by modulating eIF2α phosphorylation through its phospholipid esterase activity. Similarly, neither PTEN nor the PTENα-C297S mutant significantly influenced eIF2α phosphorylation in Pten − /− MEFs upon H_2_O_2_ exposure [54]. Instead, we identified two other major regulatory mechanisms by which PTEN loss in BMDMs promoted SG formation. First, the upregulation of the expression and transcription of the SG nucleating protein TIAL1 promotes SG assembly by lowering the threshold [55] of protein‒RNA-protein binding interactions. Second, the activation of the PAM pathway caused by PTEN dysfunction also contributed to accelerated SG formation. According to our data, the inhibition of the PI3K, AKT, or mTOR pathways led to a reduction in SG formation, indicating that the PAM pathway also plays a regulatory role in SG formation in BMDMs. In the PAM pathway, MTOR exists in both the mTORC1 and mTORC2 complexes and has different functions in regulating metabolic control throughout life [56–58]. Mammalian mTORC1 responds to various stress factors, including oxidative stress, DNA damage, and unfolded protein stress [59, 60]. Oxidative stress reportedly inhibits mTORC1 through the small protein REDD1 [61, 62]. Moreover, mTORC1 has been shown to promote SG assembly by phosphorylating and suppressing the activity of the ribosome protein S6 kinase beta-1 (p70-S6K) and eukaryotic translation initiation factor 4E-binding protein 1 (4E-BP1) [38]. mTORC1 overactivation increases the number of SGs induced by arsenate or heat stress [63]. Interestingly, SGs also inhibit mTORC1 expression in yeast and mammalian cells through various mechanisms. When mTORC1 is inhibited, the assembly of mTORC1-driven SGs may constitute negative feedback and limit mTORC1 activation by stress to help regulate cellular metabolic processes and homeostasis under stress [64, 65]. Moreover, G3BP was recently found to recruit the TSC complex to the lysosome membrane through a stress-independent mechanism, where it acts as a key protein involved in modulating mTORC1 activity [66]. PI3K and MAPK/p38 are also believed to promote SG formation via mTORC1 activation [67] in cancer cells, which is consistent with our findings that PTEN loss accelerated SG assembly through PAM pathway activation in BMDMs.

As a member of the EGR family, EGR1 (early growth response-1) is an important transcription factor widely expressed in many cell types [68]. EGR1 activation could be induced by multiple extracellular signals and mediates various physiological processes as a response to growth factors, cytokines, reactive oxygen species and other environmental stress [69–72]. It was reported that EGR1 plays a critical role in the initiation of an immune response [73]; in macrophages, a large share of EGR1 target regions are enhancers associated to the inflammatory response [74]. The PTEN/AKT pathway is reported to be linked to EGR1-mediated transcription; and EGR1 depletion stimulated PTEN expression, whereas EGR1 overexpression inhibited PTEN expressions [75]. Interestingly, there were inconsistent findings on the relationship between the expression of PTEN and EGR1, and several other studies showed a positive regulation of PTEN expression by EGR1 binding to PTEN promoters [76, 77]. In our research, we identified another role that EGR1 plays in mediating the immune response of macrophages, which is regulated by PTEN. Furthermore, our results indicated that EGR1 participates as a regulator of SG assembly in macrophages. These findings might help in deeper understanding and further research on the mechanisms of SG formation and shed light on additional functions of EGR1.

There are several limitations in this study. Our research focused on BMDMs, which are the most common type of macrophage in the stroma. Polarization is a typical characteristic of macrophages when facing various environmental features. Due to the complexity of the TME, macrophage polarization changes and is not strictly defined as M1 or M2. In our study, macrophages were stimulated with LPS and tended to act like M1 macrophages. To confirm the polarization subtype, we assessed the polarization markers of BMDMs and did not observe a transition from M1 to M2. Considering that the functions and molecular characteristics of macrophages differ among various polarization states, it is difficult to explore every macrophage subtype at once. However, additional studies of SGs in M2 macrophages and other macrophage subtypes are needed. Additionally, both phagocytosis and SG formation are dynamic processes, which makes them difficult to assess and observe, especially in vivo and in real time. Therefore, obtaining evidence of the relationship between SGs and phagocytosis in vivo is challenging; notably, the observation of G3BP1 aggregation in BRCA sections in our study is rare and valuable.

In summary, the results of our study showed that when confronted with oxidative stress, as a self-protective manner, the assembly of SGs in macrophages consumes tremendous amounts of ATP, which impairs cellular skeletal remodelling for phagocytosis. Moreover, PTEN loss in macrophages promotes SG formation by upregulating TIAL1 expression and PAM pathway activation, thus ultimately impairing antitumour immunity in the BRCA microenvironment. Our findings may lead to the development of new strategies and provide a theoretical basis for improving the treatment and outcomes of BRCA patients, especially those with insufficient myeloid PTEN function.

## Methods

### Mice

C57BL/6 J mice were purchased from Slaccas Animal Cooperation. *Pten*^f/f^ mice and *LysM-cre* mice (B6.129P2-Lyz2tm1(cre)Ifo/J) were gifts from the Laboratory of Prof. Rongbin Zhou at the University of Science and Technology of China (USTC). The two mouse genotypes were hybridized to obtain the myeloid PTEN deficiency phenotype, which was termed the Lys2 Cre *Pten*^f/f^ (*Pten*^mKO^) mouse. Mice were backcrossed for at least eight generations for purification. All the mice were specific pathogen-free (SPF), and all the mouse experiments were conducted on 10 ~ 20-week-old animals. All the procedures complied with the relevant ethical rules and regulations. This study was approved by the Ethics Committee of the University of Science and Technology of China (2021-N(A)-327).

### Human tissue samples

A total of 27 BRCA patients treated with standard neoadjuvant chemotherapy (anthracycline and taxane-based regimens and anti-Her2 targeted therapy for HER2+ patients) at the First Affiliated Hospital of USTC were included in the BRCA cohort in this study. Tumour tissues were obtained at two-time points. Biopsies before treatment were collected at diagnosis, and tissue samples for sectioning were collected during radical mastectomy. Patients underwent surgery 3-4 weeks after at least 4 rounds of standard neoadjuvant treatment. Therapeutic efficacy was determined by multiple pathologists who assessed the pathological changes among the sections at two-time points according to the Miller–Payne (MP) scoring system. The grading criteria are described in the Supplementary Table 1 Patients with an MP grade of 1-2 were classified as chemoresistant, whereas patients with an MP grade of 3-5 were classified as chemosensitive. Detailed information, including the patient number, MP grade, and MOD of specific targets, is summarized in Supplementary Table 2. All of the samples were collected after obtaining informed consent from the patients and after approval by the review board of USTC (2021-N(H)-216). The study complied with all relevant ethical regulations regarding research involving human participants.

### Reagents

Arsenite and ATP were purchased from Sigma‒Aldrich. Anisomycin was purchased from Absin (abs817944). CFSE was purchased from Invitrogen (65-0850-84). Actin-Tracker Green was obtained from Beyotime (C1033, 1:60), and Cell Tracking Dye Kit Red-Cytopainter was purchased from Abcam (ab138893). LPS was obtained from Invitrogen. The following primary antibodies were used: anti-G3BP1(Proteintech, 66486-1-Ig, 1:400(IF) or 1:5000(WB); BD Trausduction Laboratories, 611126, 1:400), anti-G3BP2 (Proteintech,16276-1-AP, 1:3000), anti-TIA1 (Proteintech, 12133-2-AP, 1:3000), anti-TIAL1 (Cell Signaling Technology, 8509 S, 1:800 for IHC and 1:1000 for WB), anti-FMR1 (Proteintech, 13755-1-AP, 1:3000), anti-EGR1 (Cell Signaling Technology, 4154 S, 1:1000 for WB and 1:50 for Chip-seq), anti-CD68 (Cell Signaling Technology, 76437 S, 1:400), anti-DDX3 (Bethyl Laboratories, A300-474A, 1:1000), anti-PTEN (Cell Signaling Technology, 9188 S, 1:1000 (for WB), and Proteintech, 66486-1-Ig, 1:200 (for IHC)), anti-MYH pan monoclonal (Elabscience, E-AB-22021, 1:100), anti-beta actin (Proteintech, 66009-1-Ig, WB 1:5000), anti-eIF2α (Cell Signaling Technology, 5324 T, 1:1000), anti-Phospho-eIF2α (Cell Signaling Technology, 3597 S, 1:1000), and FITC anti-mouse I-A/I-E Rat IgG2b (Biolegend, 107605, 1:200). Secondary antibodies, including Alexa 488 goat anti-mouse, Alexa 488 goat anti-rabbit, Alexa 594 goat anti-rabbit, Alexa 594 goat anti-mouse, peroxidase goat anti-mouse HRP, and peroxidase goat anti-rabbit HRP, were obtained from Jackson Laboratories.

### Cell culture

The 293 T cell line and L929 fibroblast line were cultured in DMEM (Gibco) supplemented with 5% newborn calf serum (Gibco), 5% FBS and 1% penicillin‒streptomycin (Beyotime Biotechnology) in an incubator at 37 °C with 5% CO_2_. The 4T1 breast cancer cell line (from BALB/c mice) was cultured in RPMI-1640 medium (Gibco) containing the same supplements as above. BMDMs were derived from the bone marrow cells (BMCs) of C57BL/6 mice. After the removal of red blood cells, BMCs were cultured in DMEM containing the same supplements as above, in addition to 10% supernatant from L929 cells. The culture medium was replaced every other day for 7 days or until the BMDMs had matured. All of the cell lines were mycoplasma-free.

### BMDM stimulation and stress granule induction

After 7 days of culture and maturation, the BMDMs were stimulated with LPS (100 ng/mL) for 4 h. BMDMs were then treated with arsenite (90 µM) for 1 h to induce stress granules. For SG inhibition, cells were treated with anisomycin (25 µg/ml) for 20 min before arsenite treatment. For PAM pathway inhibition, the cells were treated with wortmannin (100 nM), rapamycin (100 nM) or AZD5356 (3 μM) for 2 h before arsenite treatment. In the ATP supplementation experiment, ATP (4 M for 90 min) was provided before arsenite treatment. The cells were then collected for immunofluorescence analysis, coculture or flow cytometry. The cell extracts were processed for immunoblotting or ATP analysis, and the cell supernatants were processed for cytokine analysis using a CBA kit.

### Coculturing of BMDMs and 4T1 cells

4T1 cells were digested with 0.1% trypsin-EDTA, and the reaction was terminated by the addition of an antibiotic-free medium. After counting, the cells were washed with PBS and resuspended to a concentration of 1×10^6^ cells/mL. Then, 1×10^6^ cells/μL were stained with CFSE, incubated at 37 °C for 20 min and incubated on ice for 3 min to terminate staining, followed by centrifugation, a PBS wash and resuspension in complete medium. Mature BMDMs (d7) were digested, washed, counted, and resuspended in a complete medium. Tracking dye Red (25×) was added to the cells, which were then incubated at 37 °C for 30 min. After staining was complete, the BMDMs were washed with PBS and resuspended in a complete growth medium. BMDMs and 4T1 cells were mixed at a 5:1 ratio, transferred to a round-bottomed 96-well low-adhesion plate, and incubated in a 37 °C incubator for 4 ~ 6 h. Phagocytosis was observed and analysed via fluorescence microscopy or flow cytometry.

### Flow cytometry and cell sorting

For BMDM authentication, mature BMDMs (d7) were blocked at 4 °C for 5 min and then stained with antibodies against F4/80 or CD11b at 4 °C for 20 min. For MHC-II molecule detection and measurement, BMDMs were primed with LPS and pretreated with arsenite. Then, the cells were fixed and stained with anti-FITC/MHC-II (I-A/I-E) antibodies. For phagocytosis experiments, BMDMs were stimulated with LPS and pretreated with arsenite or other agents as needed. Single-cell suspensions of BMDMs were stained with Tracking dye Red (detected in the PE channel), and 4T1 cells were stained with CFSE (detected in the FITC channel). After washing, the samples were subjected to flow cytometry, and the results were processed and analysed with FlowJo (version 10.8.1). The gating strategy is described in Supplementary Fig. 2.

### Confocal microscopy and immunofluorescence

The prepared BMDMs were pretreated with PHEM, fixed with 4% paraformaldehyde (PFA) for 15 min at room temperature (RT) and washed with phosphate-buffered saline (PBS). Then, the cells were permeabilized with 0.2% Triton X-100 for 5 min and blocked with 1% BSA in TBST for 1 h. The cells were incubated with primary antibodies at 4 °C overnight, secondary antibodies at RT for 1 h and DAPI for 3 min. After all the staining procedures, Dako Fluorescence Mounting Medium (Dako North America, S302380-2) was used for mounting. Confocal microscopy analyses were carried out using a Zeiss LSM980. Images were acquired with ZEN (version 3.8) and analysed by ImageJ (version 1.8.0).

### Immunohistochemistry (IHC) of human biopsies

The human breast tumour tissue was paraffinized and cut into consecutive serial sections by pathologists. After heating in a 60 °C oven for 25 min, removing paraffin with xylene and hydrating in ethanol, the sections were heated in sodium citrate (pH 6.0) for antigen retrieval. Then, the samples were treated with 3% H_2_O_2_ in methanol_,_ blocked with goat serum and incubated with primary antibodies (anti-PTEN, 1:200; 66486-1-Ig, Proteintech; anti-CD68, 1:200; 76437 S, Cell Signaling Technology; anti-TIAL1, 1:800; 8509 S, Cell Signaling Technology). at 4 °C overnight. After washing with PBS, the sections were incubated with secondary antibodies at RT for 30 min. Then, the sections were stained with DAB and haematoxylin. Four random fields in each section were imaged for analysis. The integrated optical density (IOD) of PTEN in cells was calculated by the mean DAB intensity using ImageScope (version 12.4.6.5003), and PTEN expression was calculated as MOD = IOD/(the total area of captured cells).

### Quantitative PCR

Total RNA was extracted from the cells using the TransZol Up Kit (Transgene, ET101-01). First-strand cDNA was synthesized using a HiScript II cDNA Synthesis Kit (Vazyme, R212-02) and stored at -80 °C. Quantitative PCR was conducted using AceQ qPCR SYBR Green Master Mix (Vazyme, Q111-02). The primers used are listed in Supplementary Table 6. The program was run and data were collected with PikoReal software (version 2.2).

### ATP measurement

The S0026 ATP detection kit was used to assess intracellular ATP levels. Cell lysate was added to a 6-well plate (200 μL per well) on ice. The transferred cell lysate was centrifuged at 12,000× *g* at 4 °C for 5 min, after which the supernatant was collected. The samples or diluted standards were added to the test wells with working solution. A chemiluminescence instrument was used to measure the RLU or CPM in the wells 2 sec later. The concentration of ATP in the sample was calculated by substituting the RLU value or CPM in the sample well into the standard curve. The data were analysed with Excel and GraphPad 9.

### Cytokine analysis using the CBA method

Lyophilized standards were diluted according to the concentration gradient: undiluted/1:2/1:4/1:8/1:16/1:32/1:64/1:128/1:256/diluent and stored at 4 °C. Antibody-captured microspheres were aspirated at the required amount and mixed well. The test samples were diluted based on the cytokine concentration. Then, 50 µL of capture beads was added to the test tube, 50 µL of dilution fluid was added to the negative control tube, and 50 µL of sample was added to each remaining tube. Then, 50 µL of PE detection reagent was added to all the tubes. After vortexing and mixing, the tubes were incubated at RT in the dark for 2 h with one vortex per hour. After antibody labelling was complete, 1 mL of washing buffer was added to each test tube, and the tube was centrifuged at 200 × g for 5 min. Three hundred microlitres of washing buffer was added to each tube, after which the supernatant was discarded. The microspheres were resuspended for testing. The results were analysed by FCAP Array software.

### Dual-luciferase reporter gene assay

The plasmid used was pGL3-basic. 293 T cells were seeded in 24-well plates and transfected when the confluence reached approximately 70%. Plasmid or transfection reagent was added to 25 µl of Opti-MEM, which was subsequently incubated at RT for 5 min, gently mixed and incubated at RT for another 20 min. A total of 450 µl of serum-free and antibody-free DMEM was added to each well to obtain the transfection mixture. The medium in the 24-well plate was replaced with the transfection mixture, which was discarded after 6 h of incubation. The medium was subsequently replaced with 500 µl of complete medium per well, after which the cells were cultured for another 48 h. The medium was then discarded, and 200 µl of mixed reporter gene cell lysis solution was added to each well; the mixture was shaken on ice for 20 min and then centrifuged at 15,000×g for 5 min at 4 °C. The supernatant was retained for testing. The Renilla luciferase detection substrate (100×) and Renilla luciferase detection buffer were prepared at a ratio of 1:100 and mixed to obtain the Renilla luciferase working solution. One hundred microlitres of the sample was added to each well, and an equal volume of the reporter gene cell lysis solution was used as the blank control. The detection wavelength of the multifunctional enzyme marker was set to 560 nm. Firefly luciferase detection reagent (100 µl) was added, followed by mixing. The relative light unit (RLU) was subsequently measured (RLU1). The detection wavelength was 465 nm, and 100 µl of Renilla luciferase working solution was added for the measurement of RLU (RLU2). With Renilla luciferase set as the internal reference, the activation degree of the target reporter gene among the different samples was calculated by calculating the RLU1/RLU2 ratio.

### CHIP sequencing and qPCR

The BMDMs were cross-linked with 1% paraformaldehyde for 10 min and then quenched with glycine for 5 min. After washing twice with PBS, the fixed cells were pipet harshly to disrupt cell clusters and spin them at 1500 × g for 5 min at 4 °C. After the supernatant discarded, the samples were added with 500 µl of ChIP Lysis buffer with protease(1:20) and phosphatase inhibitors, and then sonicated for 20 min for each sample. Save one 20 µl aliquot of cell lysate from each sample as input DNA. Add 2 µl of 20 mg/ml Protease K to one aliquot of control DNA and one aliquot of input DNA. Mix and incubate at 55 °C water bath for 120 min. Spin the samples at 20,000 × g for 15 min at 4 °C to pellet debris. Collect the supernatant for IP and adjust each sample into the same concentration with ChIP Lysis buffer with protease and phosphatase inhibitors. Add 25% of spike-in chromatin into each sample with addition of 2-5 ug antibody and then incubate the mixture at 4 °C. After magnetic protein A/G beads blocking, resuspend beads in 50 µl ChIP Lysis buffer and transfer beads into cell lysate. After incubating beads with samples for 2–4 hr at 4 °C and washing with wash buffer, add 200 µl ChIP Elution Buffer into each sample and take the 20 µl input samples out from -20°C and add 180 µl of ChIP Elution Buffer into each input sample. Add 3 µl Protease K into each sample above and incubate the samples in a thermo shaker (65 °C, 1000 rpm) for DNA purification.

BMDMs from three independent mice in each group (*Pten*^f/f^ and *Pten*^mKO^) were used for the ChIP-seq. ChIP-seq libraries were constructed with ChIP and input DNA using VATHS Universal DNA Library Prep Kit for Illumina (Vazyme). Libraries were clonally amplified in a flow cell and sequenced with Illumina Hiseq 2500 (Illumina) to generate paired-end sequences. In our analyses, the alignment code is “bowtie2 -x $database/genome -1 ${id}_1.fq -2 ${id}_2.fq -S ${id}.sam -p 18 -k 6 -X 700 --local”, and the call peak code is “macs2 callpeak -t ${id}.rmdup.bam -n $id -B --trackline -g mm -s 150 -q 0.05 -f BAMPE --keep-dup all”.

In the ChIP-qPCR analyses, the values from the immunoprecipitated samples were normalized to that from the input DNA. Three pairs of primers were used for the qPCR experiments and analysis which are listed in Supplementary Table. 6 (TIAL1-primerA/B/C).

### Statistical analysis and reproducibility

The experiments were independently conducted and repeated at least twice with similar results. Statistical analyses were carried out with GraphPad Prism 8. Data are presented as the mean±s.e.m. for Fig. 1B (left),C (left), 2B, C, 4C, D, E, 5C, E, F and 6B, D, F and supplementary Fig. 1D (right); as a Kaplan-Meier survival curve for Fig. 1A and supplementary Figs. 1C, D; as Pearson correlation coefficient for Fig. 1B(right),C (right) and 2 G,H. Statistical significance was assessed by one-way ANOVA for Figs. 4C, 5C, E, F and 6B, D,F and supplementary Figs. 2A, G with multiple comparisons; by two-way ANOVA for Figs. 4D,E and 6A, with multiple comparisons; and by unpaired two-tailed Student’s t-test for Fig.1B(left),C(left) and 2B,C. For survival curves, two-sided log-rank (Mantel–Cox) tests were used. Statistical significance is displayed as *p* value, and described as *P < 0.05, **P < 0.01 and ***P < 0.001 in the corresponding figure legend.

Immunoblots for Figs. 2D, E and 3E are representative of three independent experiments with similar results. ChIP-qPCR results for Fig. 4D, E are representative of 3 independent qPCR experiments with 3 different pairs of TIAL1 primers each. ATP level measurement for Fig. 5A and B are representative of three independent experiments with similar results;for Fig. 6B is representative of 12 wells in four independent experiments with similar results. Cytokine detection results for supplementary Fig. 2A are representative of three independent experiments with similar results. Flow cytometry for Figs. 5B, C, 6C, D, E, F and supplementary Fig. 1E, 2B, C, D are representative of three independent experiments with similar results. Immunofluorescence images for Figs. 1E, 2A, 5A, D are representative of three independent experiments with similar results. Immunohistochemistry images,MOD calculating and Miller-Payne score for Figs. 1B–E, 2F, G, H are representative of results of the paraffinized section from 27 BRCA patients in the cohort. No patient samples or mice were excluded from the experiment. The mice were used for bone marrow derived macrophages(BMDMs) isolation and no blinding or sample size estimate was performed.The sample size of the patient cohort was not predetermined by the statistical methods. Data collection and analysis were not performed blind except blinded staining and blinded analysis were performed for IHC and immunofluorescence experiments.

Flow cytometry analyses and images were processed with CytExpert(version 2.4). Immunofluorescence images were processed with ImageJ, and immunohistochemical information was collected with an ImageScope (version 12.4.6.5003).

### Supplementary information

STR profiling of 4T1

supplementary information file

mycoplasma contamination test

supplementary Tables 2-5

## Data Availability

Source data are available online for Fig. 1–7, Supplementary Figs. 1 and 2 and Supplementary Tables 1 and 6. Original data of Supplementary Tables 2–5 are available in the Dryad Digital Repository (https://datadryad.org/stash/share/l2iTjI4GoCLF2pD9eZjI2r6Ib2RTeNq0MjxN9ZYN7nA). All other data supporting the findings of this study are available from the corresponding authors on reasonable request.
