# Supplementary material for: Myeloid PTEN loss affects the therapeutic response by promoting stress granule assembly and impairing phagocytosis by macrophages in breast cancer
Source: Cell Death Discov. 2024 Jul 30;10:344. doi: 10.1038/s41420-024-02094-0 (PMC11289284; doi:10.1038/s41420-024-02094-0)
Supplement: Supplementary file 1 — STR profiling of 4T1 [file 41420_2024_2094_MOESM1_ESM.pdf]

Cell Line Authentication Service

STR Profiling Report

Sample From: Division of Life Sciences and Medicine, University of Science and Technology of China, Hefei, Anhui

Sample Type: Cell Line

Testing Method: STR Genotyping

Report Time: May 19, 2024

Add: Room 502, No.2, Zhongxing Creative Park, Lane 1015, Longteng Road, Songjiang District, Shanghai / Tel:+86-18521538068

COMPANY STATEMENT

1. THIS REPORT IS ONLY RESPONSIBLE FOR THE SAMPLES ANALYZED.
2. THE TESTING RESULTS AND THE ORGANIZATION NAME WILL NOT BE USED FOR ADVERTISEMENT, COMMERCIAL EXHIBITIONS, COMMERCIAL PERFORMANCE AND OTHER COMMERCIAL ACTIVITIES.
3. OBJECTIONS SHOULD BE RAISED WITHIN FIFTEEN DAYS AFTER THE RECEIPT OF THIS REPORT.
4. THE PAPER REPORT WITH CONTENT ALTERING, ADDING OR WITHOUT THE STAMPED SEAL OF THE COMPANY ARE INVALID.

Testing Company: Shanghai Biowing Applied Biotechnology Co. Ltd

Address: Room 502, NO.1015 Longteng Rd, Songjiang District, Shanghai

Tel: +86-18521538068

Contact: Shuangning Zhu

E-mail: zhusn@biowing.com.cn

Cell Line Authentication – STR Profiling Report

Sample code

Table 1. Sample Code

Customer's code	Company Code
4T1	20240517-01

Sample Number:1

Sample Type: Cell line

Testing Type: STR

Testing Method:

DNA was extracted by a commercial kit from CORNING (AP-EMN-BL-GDNA-250G). The ten STRs including one human locus were amplified by multiplex PCR and separated on ABI 3730XL Genetic Analyzer. The signals were then analyzed by the software GeneMapper..

Data Interpretation:

Cell lines were authenticated using Short Tandem Repeat (STR) analysis asdescribed in 2021 in ANSI Standard (ASN-0002) by the ATCC Standards Development Organization (SDO) and in Capes-Davis et al., Match criteria for human cell line authentication: Where do we draw the line? Int J Cancer.2013;132(11):2510-9.

DSMZ tools was used to carry on the cell line comparison, which contains 2455 cell lines STR data from ATCC, DSMZ, JCRB, ECACC, GNE, RIKEN and EXPASY databases. If the cell is not included in the above cell library, users need to compared with other databases.

Add: Room 502, No.2, Zhongxing Creative Park, Lane 1015, Longteng Road, Songjiang District, Shanghai / Tel:+86-18521538068

Test Results

1. STR profile

Table 2. STR and Amelogenin Genotyping Results of Cell line.

Loci	Sample information				Cell Bank information		
	Sample name：4T1				Cell line name：4T1		
	Allele1	Allele2	Allele3	Allele4	Allele1	Allele2	Allele3
18-3	19				19		
4-2	21.3				21.3		
6-7	12				12		
19-2	13				13		
1-2	17				17		
7-1	25.2				25.2		
8-1	13				13		
1-1	15	16			15	16	
3-2	14	15			14	15	
2-1	16	17			16	17	
15-3	22.3				22.3		
6-4	18				18		
13-1	16.2				16.2		
11-2	18	19	20		18	19	20
17-2	15				15		
12-1	16				16		
5-5	14				14		
X-1	25				25		
TH01							
D4S2408							

Add: Room 502, No.2, Zhongxing Creative Park, Lane 1015, Longteng Road, Songjiang District, Shanghai / Tel:+86-18521538068

2. database annotation

Figure 1. STR matching analysis

Accession	Name	Nº Markers	Score	STR 1-1	STR 1-2	STR 2-1	STR 3-2	STR 4-2	STR 5-5	STR 6-4	STR 6-7	STR 7-1	STR 8-1	STR 11-2	STR 12-1	STR 13-1	STR 15-3	STR 17-2	STR 18-3	STR 19-2	STR X-1
NA	Query	NA	NA	15,16	17	16,17	14,15	21.3	14	18	12	25.2	13	18,19, 20	16	16.2	22.3	15	18,19	13	25
CVCL_0125	4T1	18	100.0 0%	15,16	17	16,17	14,15	21.3	14	18	12	25.2	13	18,19, 20	16	16.2	22.3	15	18,19	13	25

Note: The STR online match analysis of the test cell against EXPASY database, showing cell number (Cell No.) and cell name.

3. Authentication

☐ The submitted sample profile is mouse, but not a match for any profile in the DSMZ.

☑ The submitted profile is an exact match for the following human cell line(s) in the DSMZ STR database (8 core loci plus Amelogenin):**4T1**.

☐ The submitted profile is similar to the following DSMZ human cell line: /.

- **Note:** A cell line can considered to be authenticated when 80% (exact match) of the alleles in its STR profile match profiles from tissue or other cell line samples from that donor or from database. Cell lines with between a 55% to 80% (similar) match require further profiling for investigation of relatedness.

Appendix:

1. Genotyping Strategy and Site Distribution

Table S1. Experimental Strategy and Sites

	方案 1	方案 2	方案 3	方案 4
**1**	18-3	1-2	2-1	TH01
**2**	4-2	7-1	15-3	D5S818
**3**	6-7	8-1	6-4	17-2
**4**	19-2	1-1	13-1	12-1
**5**		3-2	11-2	5-5
**6**				X-1

The allele match algorithm compares the 18 core loci only, D5S818, TH01 is a human site, which is used to detect whether the cell is contaminated by human sources.

Technician: Jianan Zhang

Checked by: Chenqian Zhang

Issued by: Yang Bai

Issue date: May 19, 2024

Add: Room 502, No.2, Zhongxing Creative Park, Lane 1015, Longteng Road, Songjiang District, Shanghai / Tel:+86-18521538068

Figure 2. STR profiles of sample cell line

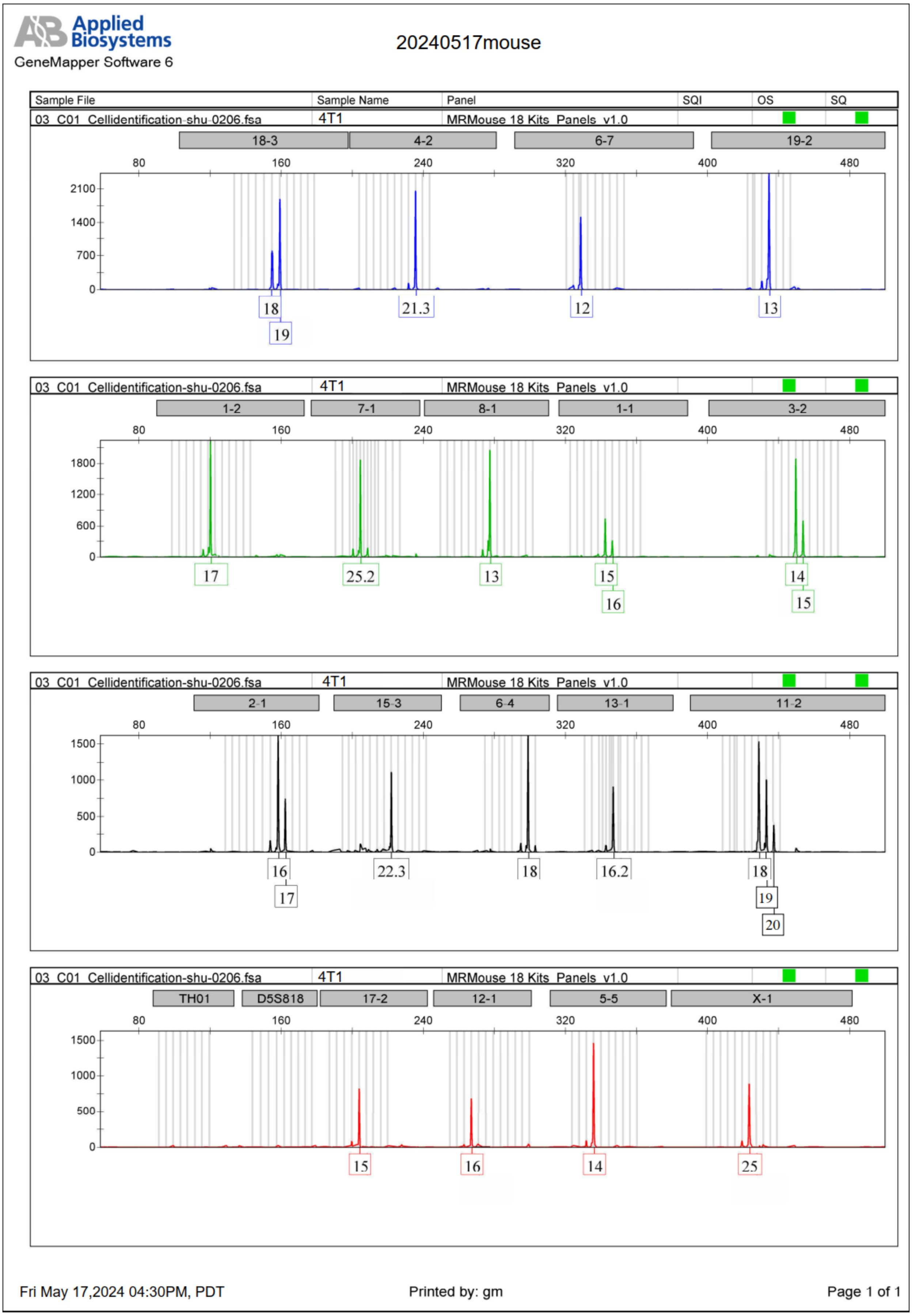

Add: Room 502, No.2, Zhongxing Creative Park, Lane 1015, Longteng Road, Songjiang District, Shanghai / Tel:+86-18521538068
