# Supplementary material for: Myeloid PTEN loss affects the therapeutic response by promoting stress granule assembly and impairing phagocytosis by macrophages in breast cancer
Source: Cell Death Discov. 2024 Jul 30;10:344. doi: 10.1038/s41420-024-02094-0 (PMC11289284; doi:10.1038/s41420-024-02094-0)

Supplementary Figure 1

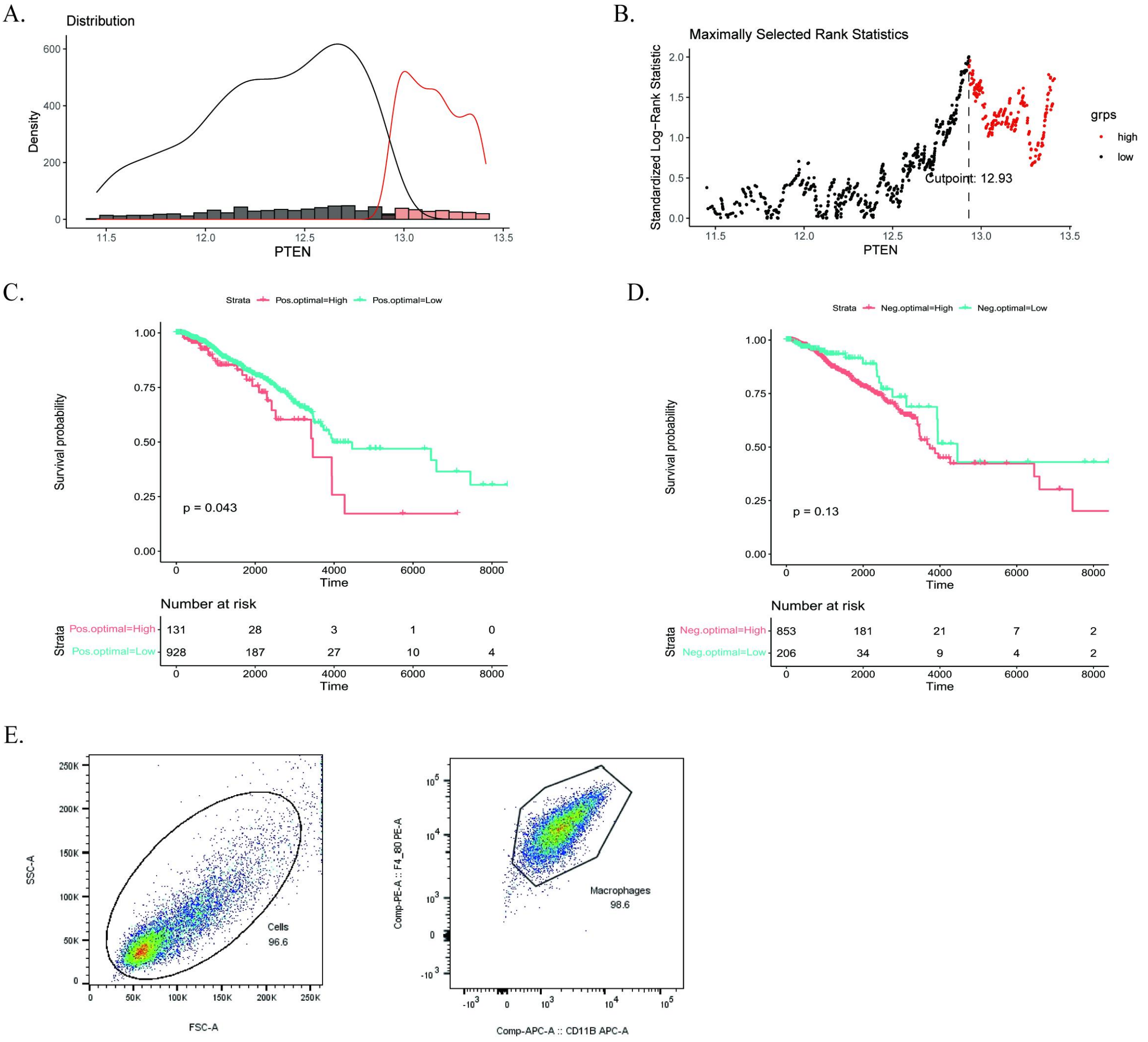

Supplementary Figure 1. Survival analysis of PTEN and oxidative stress regulatory factors in breast cancer patients. **(A)&(B)** Optimal cutoffcalculating BRCA patients data including total survival and PTEN expression were collected from TCGA-BRCA database, and optimal cutoff was calculated by R language survey package. The cutpoint was 12.93. **(C)** Overall survival analysis of BRCA patients with different level of oxidative stress response regulation. BRCA patientsd data from TCGA-BRCA database was collected and performed enrichment analyses with "positive_regulation_of_response_to_oxidative_stress"(GO:1902884) from the GSEA database. Kaplan-Meier survival analysis was performed on the classified groups: positive_regulation_high and positive_ regulation_low. **(D)** Kaplan-Meier survival analysis was performed on the classified groups: negative_regulation_high and negative_ regulation_low.((GO:1902883)) Same as (C). **(E)** Authentication of BMDMs (BMC on d7) using flow cytometry.

Supplementary Figure 2

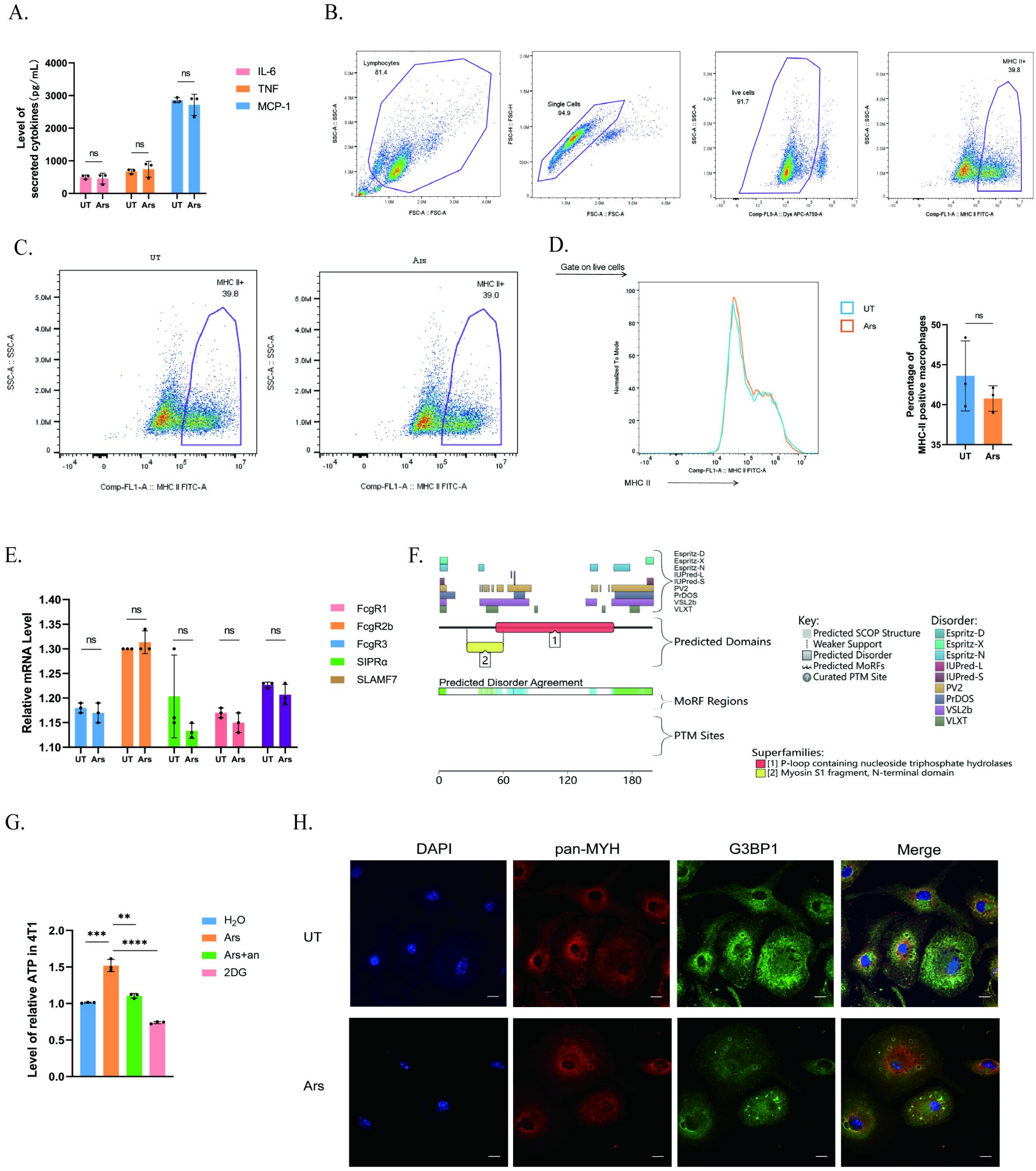

sFig. 2 Mechanisms of SGs impairing anti-tumor immunity of PTEN deficient BMDMs. LPS (100 ng/mL) was treated to the two groups of BMDM for 4 hours for activation, and the UT group was the untreated control group. The Ars group was induced by sodium arsenite at 90 μM for 1 hour. **(A).** The levels of IL-6, TNF, and MCP-1 in the cell supernatant were detected using the CBA method simultaneously. The statistical method was mean ± standard deviation; n = 3 independent experiments,double-tailed student T-test, and ns had no statistical significance. **(B)** Gating strategies sorting MHC-II positive cells. **(C and D)** The FITC-MHC -II antibody was used to mark each group of BMDM, and the percentage of positive cells in each group was calculated. **(E)** Relative mRNA level of several canonical phagocyte-related receptors on macrophages.Cells were harvested with or without SGs induction. **(F)** The IDRs prediction of MYH9 by the D2P2 database (Database of Disordered Protein Predictions).The target region were showed in red frame and highlighted in bright green. **(G)** Intracellular ATP level in 4T1 cells. 2DG was given at 5 mM for 1 hour. means ± SDs; n = 10 holes of 3 independent experiments, p value was determined by two-tailed unpaired Student's t test with multiple comparisons test. **(H)** Fluorescence staining of cytoskeleton and stress granules.Actin-Tracker Green (green) was used to label myosin filaments F-actin, and Alexa Fluor®594 (red) was used to label G3BP1 as a representative of core proteins of stress granules. The dotted accumulation of G3BP1 represented the formed stress granules . The arrow indicated the cells forming stress granules. The scale bar was 10 μm.

Supplementary Table 1

Miller-Payne (MP) scoring system

Grade(score)	Description
1	no change or some alteration to individual malignant cells but no reduction in overall cellularity
2	a minor loss of tumour cells but overall cellularity still high; up to 30% loss
3	an estimated 30% - 90% reduction in tumour cells
4	a marked disappearance of tumour cells such that only small clusters or widely dispersed individual cells remain; more than 90% loss of tumour cells
5	no malignant cells identifiable in sections from the site of the tumour; only vascular fibroelastotic stroma remains often containing macrophages. Ductal carcinoma in situ (DCIS) may be present.

Supplementary Table 6

Primers for PCR and qPCR

Gene	Direction	Sequence（5'-3'）
Actin	forward	GGCTGTATTCCCCTCCATCG
	reverse	CCAGTTGGTAACAATGCCATGT
PTEN	forward	TGGATTCGACTTAGACTTGACCT
	reverse	GCGGTGTCATAATGTCTCTCAG
FcγR I	forward	AGGTTCCTCAATGCCAAGTGA
	reverse	GCGACCTCCGAATCTGAAGA
FcγR IIb	forward	ATGGGAATCCTGCCGTTCCTA
	reverse	CCGTGAGAACACATGGACAGT
FcγR III	forward	CAGAATGCACACTCTGGAAGC
	reverse	GGGTCCCTTCGCACATCAG
FcγR IV	forward	ATGTGGCAGCTACTACTACCA
	reverse	ACCCACTTGGGGTCTAGGTTC
SIRPα	forward	AGTCACGGGGAAAGAACTGAA
	reverse	CGGCTTTGCCTACTCCTCTG
SLAMF7	forward	AGAACGCAGACTATGACACAATC
	reverse	AGGGAGCTGGGACTCTTTACC
PTEN loxp	forward	CTCCTCTACTCCATTCTTCCC
	reverse	ACTCCCACCAATGAACAAAC
Lyz2 cre	Common	CTTGGGCTGCCAGAATTTCTC
	Wt	TTACAGTCGGCCAGGCTGAC
	Mut	CCCAGAAATGCCAGATTACG
TIAL1-primerA	forward	ACACATAATCCCAGCACTAGG
	reverse	ACACATAATCCCAGCACTAGG
TIAL1-primerB	forward	GGCAGGATCCTACTTCAAGTTC
	reverse	TGAAGATTGATCACGTTGGGG
TIAL1-primerC	forward	TGGCACATACACATAATCCCA
	reverse	CAGCATGCAAGGGATGGA

Original file of Western Blot

Fig.2D

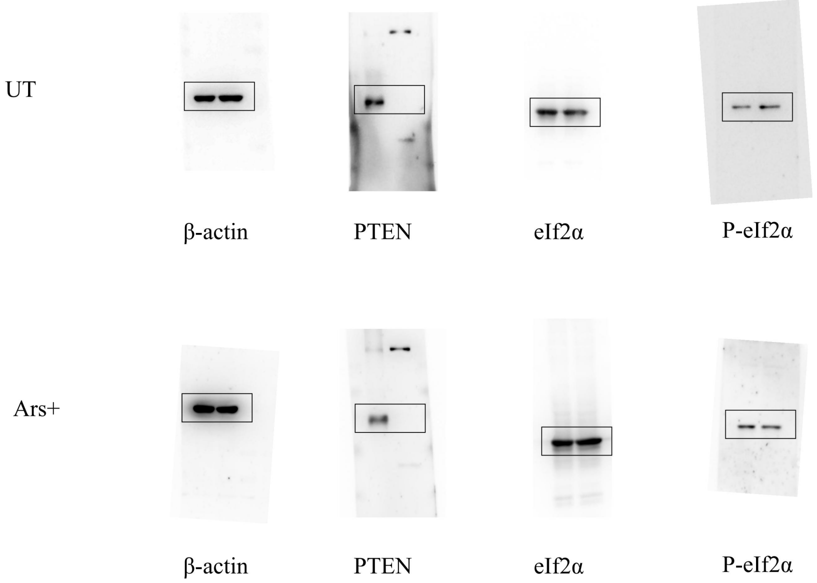

Fig.2E

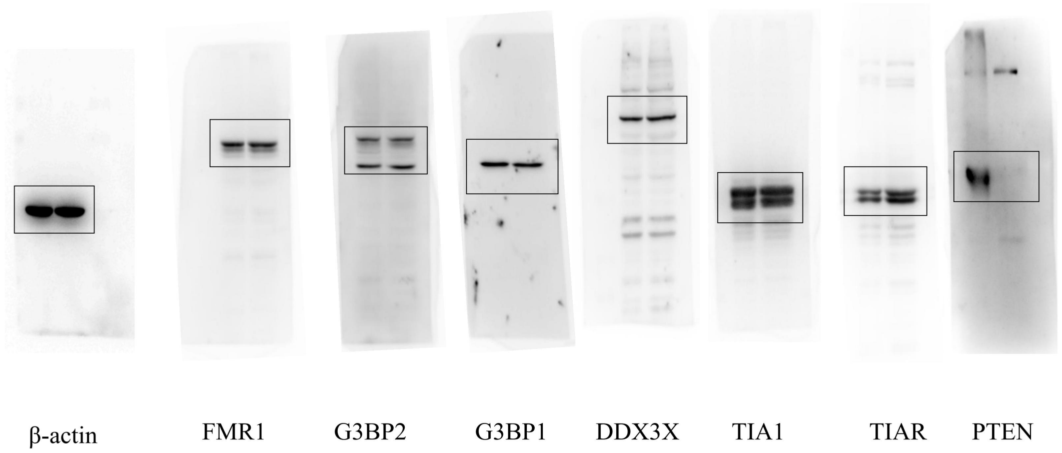

Fig.3E

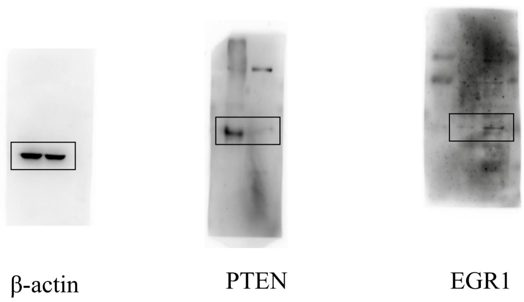

Supplement: Supplementary file 2 — supplementary information file [file 41420_2024_2094_MOESM2_ESM.pdf]
