# Supplementary figures and images for: Myeloid PTEN loss affects the therapeutic response by promoting stress granule assembly and impairing phagocytosis by macrophages in breast cancer
Source: Cell Death Discov. 2024 Jul 30;10:344. doi: 10.1038/s41420-024-02094-0 (PMC11289284; doi:10.1038/s41420-024-02094-0)

## Slide 1
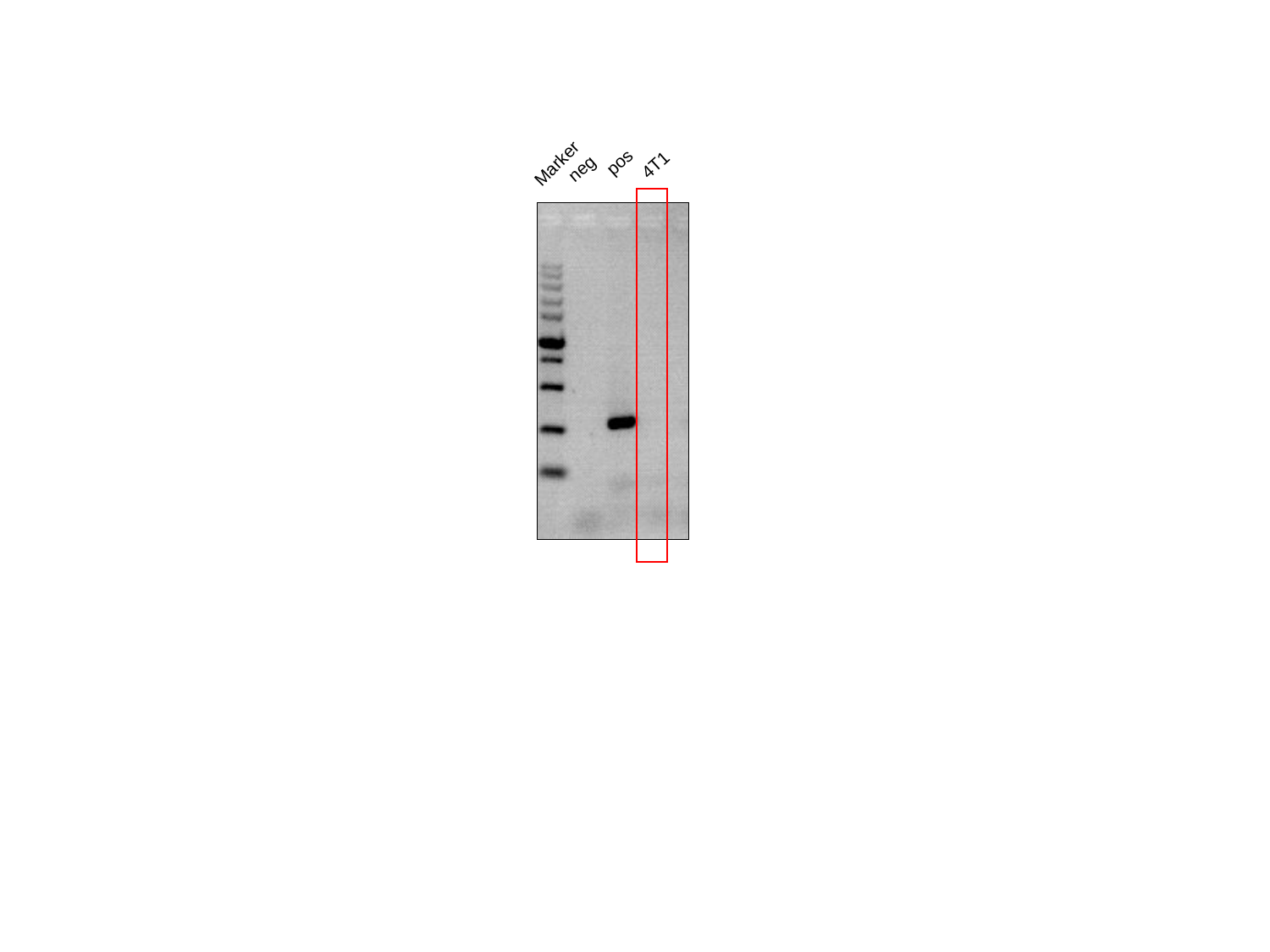

pos
4T1
Marker
 neg

Supplement: Supplementary file 3 — mycoplasma contamination test [file 41420_2024_2094_MOESM3_ESM.pptx]
